# Red and Blue Light Differently Influence *Actinidia chinensis* Performance and Its Interaction with *Pseudomonas syringae* pv. *Actinidiae*

**DOI:** 10.3390/ijms232113145

**Published:** 2022-10-29

**Authors:** Cristiana Correia, Federico Magnani, Chiara Pastore, Antonio Cellini, Irene Donati, Giuseppina Pennisi, Ivan Paucek, Francesco Orsini, Elodie Vandelle, Conceição Santos, Francesco Spinelli

**Affiliations:** 1 Department of Agricultural Sciences, Alma Mater Studiorum University of Bologna, Viale Fanin 46, 40127 Bologna, Italy; 2 IB2Lab, LAQV-Requimte, Department of Biology, Faculty of Sciences, University of Porto, Rua Campo Alegre, 4169-007 Porto, Portugal; 3 Department of Biotechnology, University of Verona, 37134 Verona, Italy

**Keywords:** bacterial canker of kiwifruit, disease control, greenhouse cultivation, LED lighting, plant–pathogen interaction, plant performance, virulence modulation

## Abstract

Light composition modulates plant growth and defenses, thus influencing plant–pathogen interactions. We investigated the effects of different light-emitting diode (LED) red (R) (665 nm) and blue (B) (470 nm) light combinations on *Actinidia chinensis* performance by evaluating biometric parameters, chlorophyll a fluorescence, gas exchange and photosynthesis-related gene expression. Moreover, the influence of light on the infection by *Pseudomonas syringae* pv. *actinidiae* (Psa), the etiological agent of bacterial canker of kiwifruit, was investigated. Our study shows that 50%R–50%B (50R) and 25%R–75%B (25R) lead to the highest PSII efficiency and photosynthetic rate, but are the least effective in controlling the endophytic colonization of the host by Psa. Monochromatic red light severely reduced ΦPSII, ETR, Pn, TSS and photosynthesis-related genes expression, and both monochromatic lights lead to a reduction of DW and pigments content. Monochromatic blue light was the only treatment significantly reducing disease symptoms but did not reduce bacterial endophytic population. Our results suggest that monochromatic blue light reduces infection primarily by modulating Psa virulence more than host plant defenses.

## 1. Introduction

Plants depend on light for their growth and thus evolved several mechanisms to adapt to changing light conditions, optimize light interception and resist light-dependent stressors [1,2,3]. In addition to different classes of pigments specialized in capturing specific light wavelength [4], plants have also developed a diverse range of photoreceptors that, directly or indirectly, regulate plant metabolism [5]. Photoreceptors enable plants to perceive light in different spectral ranges. In *Arabidopsis thaliana*, phytochromes (PHYA-E) perceive red/far-red lights (600–750 nm), cryptochromes (CRY1-3), phototropins (PHOT1-2) and F-box containing Flavin binding proteins (e.g., ZTL/FKF1, FKF1/LK andP2) sense from blue to UV-A light, and UVR8 perceive UV-B light [6].

In recent years, the development of highly efficient and low-cost light-emitting diodes (LEDs) has allowed the adoption of this technology for protected crop production [7]. However, crop responses to different light intensity, spectrum and light regimes (e.g., pulsed or continuous illumination) have not been yet fully elucidated [8,9,10]. 

Chlorophylls’ absorption peaks correspond to the blue and red spectral regions, and therefore several ratios of red and blue lights have been widely tested for yield and quality improvement in several horticultural crops [11,12,13], as well as in terms of resources use efficiency [14]. Plants acclimate to the light quality by adjusting their morphologic, anatomic and biochemical features, including photosynthesis, to optimize light capture and CO_2_ uptake [15,16,17,18]. Blue light strongly affects leaf expansion and stomatal opening and frequently promotes the development of more efficient photosynthetic apparatus when compared to red light [19,20]. Red light, on the other hand, promotes seed germination and growth and may enhance chlorophyll accumulation [21,22,23]. Using monochromatic light was shown to induce excessive shoot elongation, low chlorophyll levels and reduced photosynthetic capacity [20,21]. The combination of both blue and red light promoted net photosynthesis rate, chlorophyll content and plant dry weight in species like rice [24], spinach [25], cucumber [26] and sweet basil [27].

Light quality plays also an important role in plant defense against pathogens, either by modulating plant defenses [28,29] or interfering with pathogen virulence traits [29,30]. Light perceived by photoreceptors such as PHYA, PHYB and CRY can modulate hormone-mediated pathways, including ethylene and auxin [2], and are essential for systemic acquired resistance and hypersensitive response processes [31,32]. For these reasons, in the dark, plants seem to weaken their responses against pathogens, leading to a higher incidence of infection at night [33]. In addition, pathogens further exploit this weakness. For instance, *Pseudomonas syringae* pv. *actinidiae* (Psa) can induce plant ethylene emission, which acts as an accessory toxin, following a circadian cycle, with the peak emission in the dark [34]. However, light influence on plant disease resistance/pathogen attack is also dependent on the spectral quality and the specific pathosystem involved. Accordingly, limited information is to date available on which are the optimal light spectral properties for improved plant protection. For example, blue LED light was shown to inhibit the development of *Botrytis cinerea* in tomato plants [35], while in broad beans a similar effect was obtained with red light [36]. Light was also shown to modulate a pathogen’s virulence, altering the biofilm formation or motility [37,38]. In particular, a light-induced effect was already demonstrated for *P. syringae* pv. *tomato* [39,40], where blue light up-regulated genes related with, e.g., secretion system genes, and red light down-regulated coronatine biosynthesis-related genes, thus negatively influencing stomatal opening and pathogen entry [40]. It can therefore be assumed that light is pivotal in the plant–pathogen interaction through the regulation of both host defenses and bacterial virulence [41].

The Gram-negative bacterium *Pseudomonas syringae* pv. *actinidiae* is the causal agent of one of the most destructive diseases in *Actinidiae* orchards, the bacterial canker. Disease symptoms include brown leaf spots with chlorotic haloes, brown discoloration of buds and cankers with exudates on trunks and twigs [42]. Currently, control of the kiwifruit bacterial canker is primarily based on extensive use of Cu-based agrochemicals, and, where authorized, antibiotics. These strategies have however resulted in the development of antibiotic or Cu resistance in Psa populations [43,44]. Finding an efficient and sustainable control strategy is challenging, and, in this context, exploiting the influence of light and circadian rhythms on both host and pathogen biology and interaction represents a promising approach. While light treatments could be applied to kiwifruit orchards by using photoselective nets or plastic tunnels and in nurseries by providing specific wavelengths supplementation via LED lighting, it is important to evaluate the impact of light quality on both healthy and infected plant photosynthetic performance and, simultaneously, on disease symptom and progression. To this end, the assessment of photosynthetic performance has a two-fold aim. First, it allows for the evaluation of photo-assimilate accumulation and translocation, which are crucial not only for plant development and fruit quality, but also for inducible defenses [45]. In addition, it allows for the dissection of the physiological effects of the infection. For instance, when *Arabidopsis thaliana* and *Phaseolus vulgaris* were infected by *P. syringae*, a decreased maximum PSII quantum yield (F_v_/F_m_) [46] and photochemical efficiency of PSII (ΦPSII) [47] were observed, respectively.

Psa infection severely influences host metabolism. In infected *Actinidia* plants, an increase in the abundance of proteins participating in basal defense, pathogenesis, oxidative stress and signaling events was observed [48], together with a down-regulation of the expression of several photosynthesis-related genes [49]. Aligned with the recent data obtained with other *P. syringae* complex strains, the Psa–*Actinidia* pathosystem is hypothesized to be modulated by light quality [39,40]. Accordingly, in the present research, the effect of red and blue light on Psa–*Actinidia* interactions was investigated. Potted plants of *A. chinensis* var. *deliciosa* ‘Hayward’ were grown under LED illumination treatments featuring different red and blue combinations and inoculated with Psa. The effect of light on plant growth, photosynthetic metabolism and physiological responses to Psa was evaluated.

## 2. Results

### 2.1. Effect of Light Treatment on the Growth of Uninfected or Infected Plants

**Figure 1:**
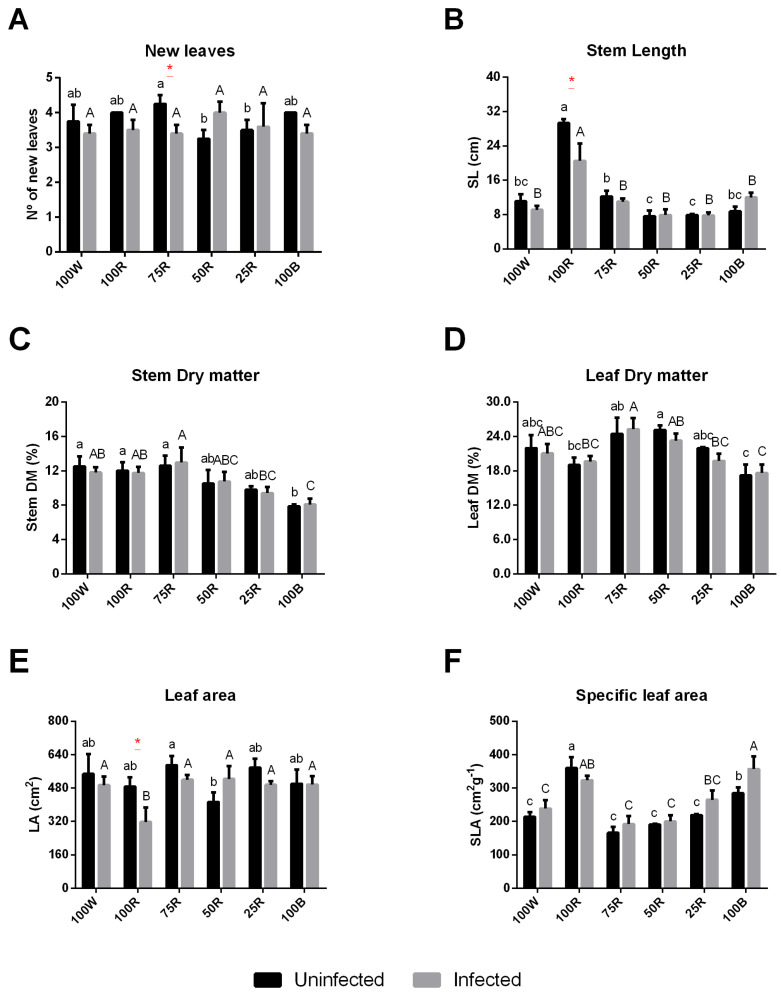
Effect of light quality on *Actinidia chinensis* var. *deliciosa* ‘Hayward’ biometric parameters and biomass. New leaves (**A**), Stem length (SL) (**B**), Stem dry matter (DM) (**C**), Leaf DM (**D**), Leaf area (LA) (**E**), Specific leaf area (SLA) (**F**). The values are presented as the mean of at least 3 biological replicates ± SE. Lowercase letters represent multiple comparisons of uninfected groups, capital letters are multiple comparisons of infected groups and asterisks are comparisons of uninfected and infected groups in the same light treatment. Mean values with the same letter or without asterisk are not significantly different (Fisher LSD, *p* < 0.05).

In uninfected plants, the number of leaves was significantly higher in plants grown under 75R as compared with those grown under 100 W, while it was reduced under 50R and 25R. In infected plants, no significant difference in leaf number was observed across light treatments (Figure 1A). Regarding the stem length (SL), 100R led to plant etiolation, in both uninfected and infected plants, compared with other light conditions. Under this light treatment, however, infected plants were significantly shorter than uninfected ones (Figure 1B). The stem %DM tended to decrease with the increase of blue light ratio, showing the same profile in uninfected and infected plants (Figure 1C). Concerning leaf indicators, 75R and 50R led both to the highest values of leaf %DM, independently from the plant health status, while the leaf area (LA) was lower only with 50R. Finally, the specific leaf area (SLA) was significantly higher under 100R followed by 100B, without any visible effect associated with Psa infection (Figure 1D–F).

### 2.2. Effect of Light Treatment on Chl a Fluorescence and Pigment Content of Uninfected or Infected Plants

**Figure 2:**
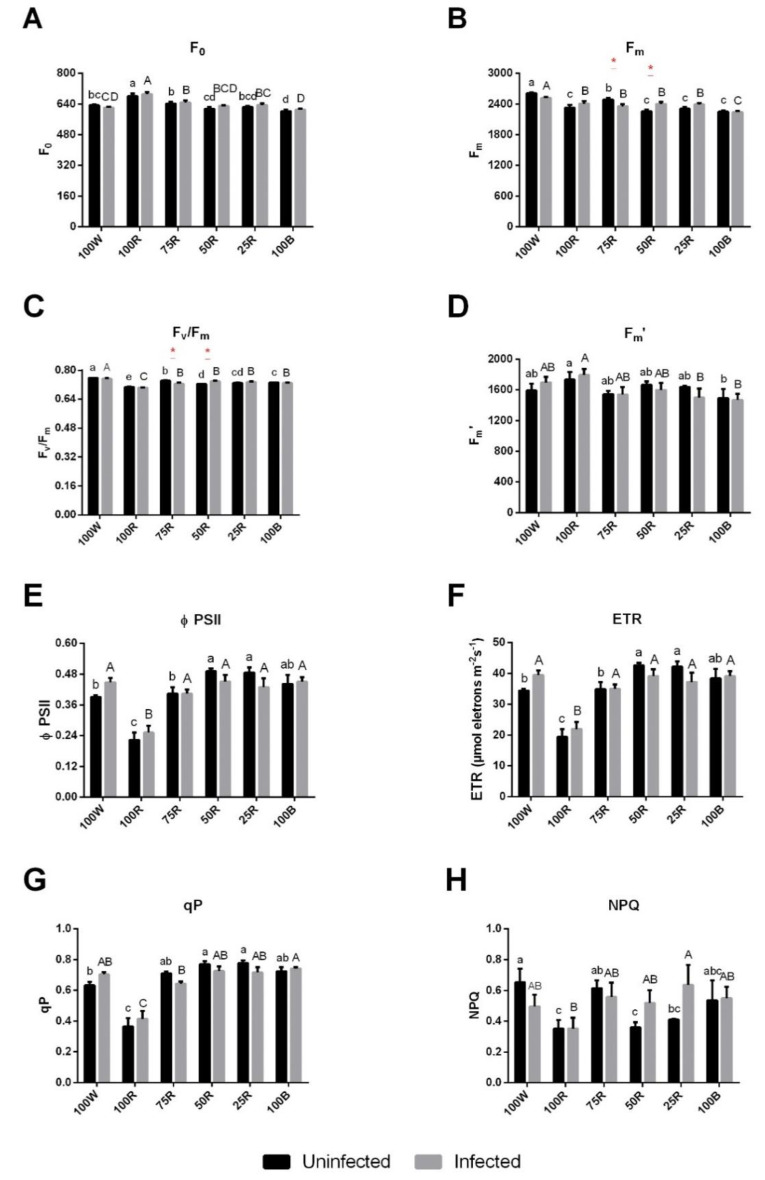
Effect of light quality on Chlorophyll a fluorescence. Minimal fluorescence yield of dark-adapted leaves, F_0_ (**A**); maximum fluorescence of dark-adapted leaves, F_m_ (**B**); maximum quantum efficiency of PSII, F_v_/F_m_ (**C**); maximum fluorescence of light-adapted leaves, F_m_’ (**D**); effective efficiency of PSII, ΦPSII (**E**); Electron transport rate, ETR (**F**); photochemical quenching, qP (**G**) and non-photochemical quenching, NPQ (**H**). The values are presented as the mean of at least 3 biological replicates ± SE. Lowercase letters represent multiple comparisons of uninfected groups, capital letters are multiple comparisons of infected groups and asterisks are comparisons of uninfected and infected groups in the same light treatment. Mean values with the same letter or without asterisk are not significantly different (Fisher LSD, *p* < 0.05).

In dark-adapted leaves, fluorescence parameters were influenced by the light treatments imposed on the plants. Uninfected and infected plants subjected to 100R light showed an increase in the initial fluorescence (F_0_), compared to the other conditions (Figure 2A). On the other hand, all light treatments significantly reduced the maximum fluorescence (F_m_) and the F_v_/F_m_ ratio, compared with the reference 100 W (Figure 2B,C). Conversely, Fm’ values differed only between 100R and 100B plants, with no effect related to plant infection (Figure 2D). ΦPSII, ETR and qP showed similar patterns, with 100R-subjected plants presenting a strong decrease compared to plants under 100 W, while 50R, 25R and 100B lights had the opposite effect, i.e., increased these three parameters. NPQ was maximum under 100 W, showing a trend to decrease under 100R, 50R and 25R (Figure 2H). The effect of the infection on the different parameters generally remained negligible (Figure 2E–H).

**Figure 3:**
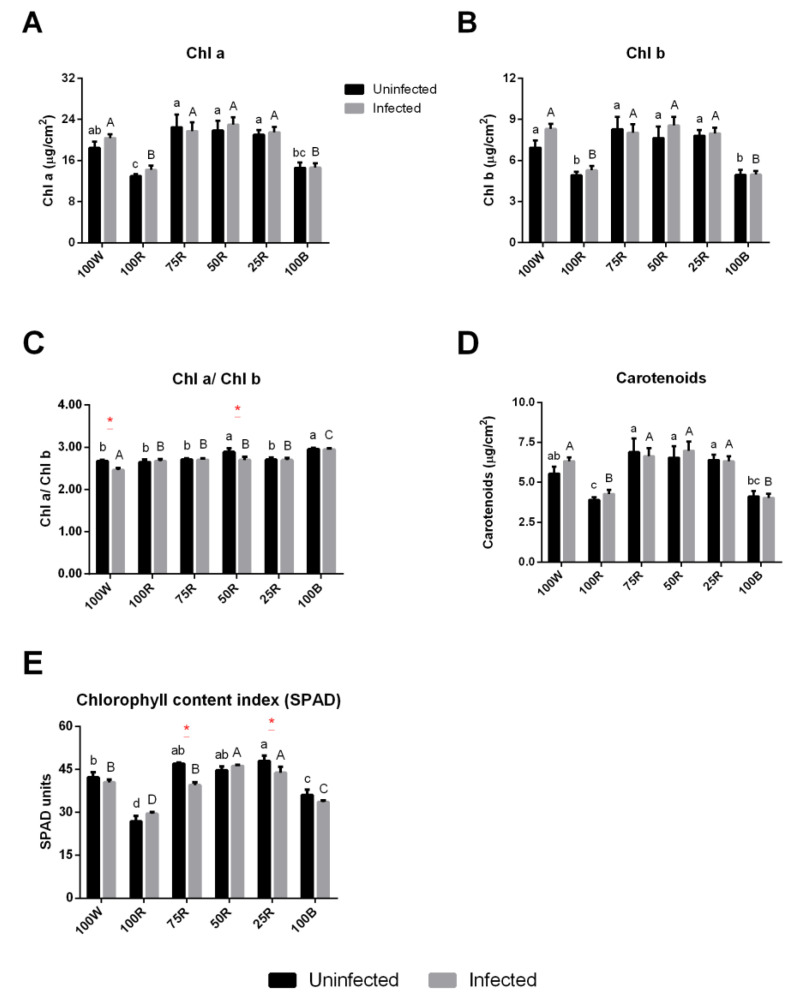
Effect of light quality on pigment content. Chlorophyll a (**A**) and b (**B**) (Chl a and Chl b) (mg cm^−2^); chlorophyll ratio a/b (Chl a/Chl b) (**C**); carotenoids (mg cm^−2^) (**D**); chlorophyll content index (SPAD) (**E**). The values are presented as the mean of at least 3 biological replicates ± SE. Lowercase letters represent multiple comparisons of uninfected groups, capital letters are multiple comparisons of infected groups and asterisks are comparisons of uninfected and infected groups in the same light treatment. Mean values with the same letter or without asterisk are not significantly different (Fisher LSD, *p* < 0.05).

Regarding the main pigments, plants under 100R and 100B lights showed a similar significant decrease of Chl a and Chl b, as well as carotenoid contents, while all R:B combinations increased the contents of these pigments (Figure 3A,B,D). Both Chl a and Chl b were modulated in the same manner displaying both graphs a similar profile. However, plants subjected to 50R and 100B displayed a significantly higher ratio in comparison to 100 W. The infection reduced the Chl a/Chl b ratio only in plants exposed to 100 W and 50R, compared to uninfected plants. Moreover, following infection, the ratio was higher in plants exposed to 100B compared to all other conditions (Figure 3C). Measurements done with SPAD showed a similar trend as observed for Chl a and Chl b contents, with minimum values obtained in plants subjected to 100R followed by 100B. Except for the 100R and 50R, SPAD values tended to decrease with the infection, but such differences were statistically significant only in plants treated with 75R and 25R (Figure 3E).

### 2.3. Rapid Light Curves (RLC)

**Figure 4:**
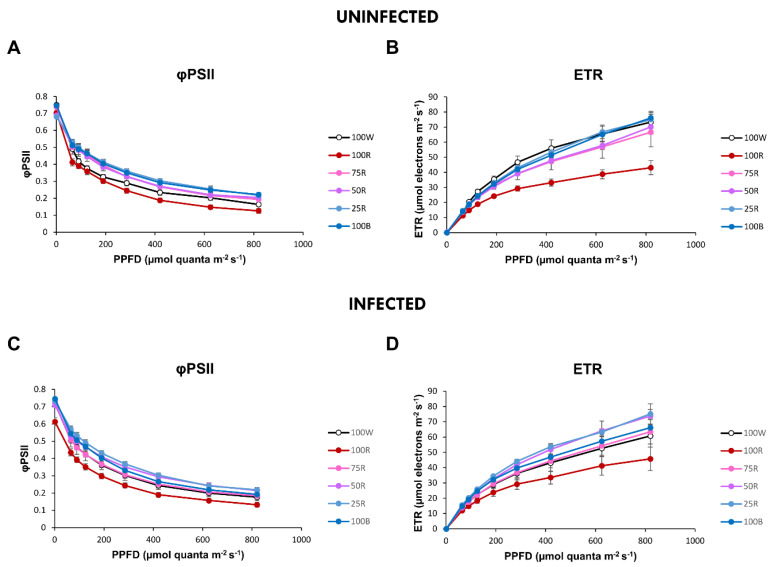
Effect of light quality on ΦPSII (**A**,**C**) and ETR (**B**,**D**) response to photosynthetic photon flux density (PPFD) on the uninfected and infected groups, respectively. The values are presented as the mean of 4–5 biological replicates ± SE. For statistical analysis see Supplementary Table S1.

Regarding the influence of the light quality on the response of ΦPSII (Figure 4A,C) and ETR (Figure 4B,D) to the photosynthetic photon flux density (PPFD), in general, 100R treated plants showed the lowest rates of photochemistry. The ΦPSII in 100R is statistically lower than in the other light treatments for light intensities up to 285 µmol m^−2^ s^−1^ (Supplementary Table S1), independent of plant health status (Figure 4A,C). Regarding the other conditions, 25R and 100B generally showed higher values than 100 W. 100R exposed plants showed a clearly different light response compared with other treatments, with a much lower electron transport capacity for light intensities higher than 65 µmol m^−2^ s^−1^ (Figure 4B,D). Overall, a small percentage of blue light (75R) was apparently sufficient for plants, which showed ETR values close to the control (100 W).

### 2.4. Gas Exchange

**Figure 5:**
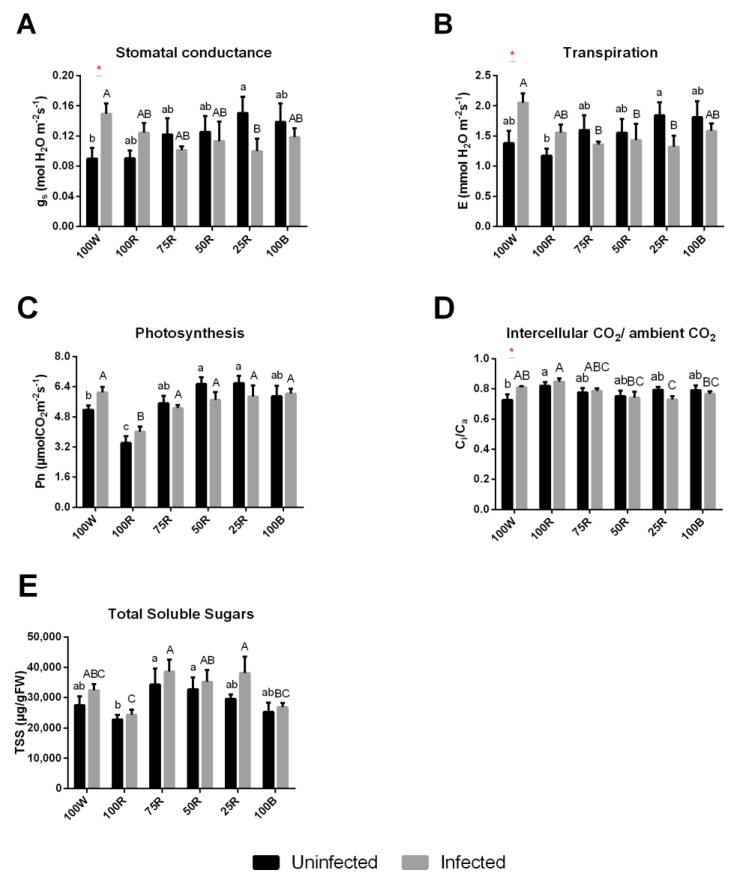
Light quality effect on leaf gas–exchange. Stomatal conductance, g_s_ (**A**); transpiration rate, E (**B**); net photosynthetic rate, Pn (**C**); intercellular CO_2_/ambient CO_2_, C_i_/C_a_ (**D**); total soluble sugars (**E**). The values are presented as the mean of at least 3 biological replicates ± SE. Lowercase letters represent multiple comparisons of uninfected groups, capital letters are multiple comparisons of infected groups and asterisks are comparisons of uninfected and infected groups in the same light treatment. Mean values with the same letter or without asterisk are not significantly different (Fisher LSD, *p* < 0.05).

In healthy plants, blue light supplementation generally increased stomatal conductance (g_s_) and transpiration rate (E), while plants under 100 W and 100R showed the lowest values. In infected plants, the values of both parameters in the control (100 W) and 100R treatments increased in comparison to uninfected plants, whereas they decreased in all conditions with supplemented blue light (Figure 5A,B). Concerning net photosynthetic rate (Pn), 100R showed the lowest value and 50R and 25R reached the highest values, which were significantly different from 100 W (Figure 5C). The influence of the infection on this parameter was negligible. C_i_/C_a_ was increased in plants exposed to 100R and reduced in 100 W. An increase in this parameter was observed in infected plants in comparison to the healthy one at 100 W and 100R, and showed a tendency to decrease under light conditions with higher percentage of blue (Figure 5D).

Concerning the total soluble sugars, the lowest values were observed with 100R, and the infection had no significant influence, despite a tendency to increase.

### 2.5. Gene Expression

**Figure 6:**
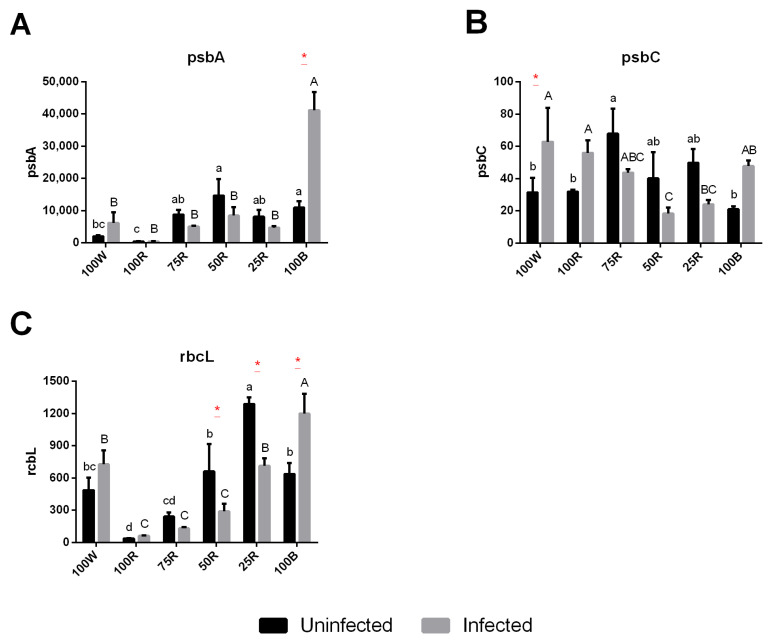
Effect of light quality on the regulation of photosynthesis-related genes. Relative expression of two core proteins of PSII (*psbA* (**A**) and *psbC* (**B**)) and the large subunit of RuBisCO (*rbcL* (**C**)). The values are presented as the mean of 3 biological replicates ± SE. Lowercase letters represent multiple comparisons of uninfected groups, capital letters are multiple comparisons of infected groups and asterisks are comparisons of uninfected and infected groups in the same light treatment. Mean values with the same letter or without asterisk are not significantly different (Fisher LSD, *p* < 0.05).

In healthy plants, the gene coding for D1 protein subunit of the PSII (*psbA*) showed a significant upregulation in all treatments supplemented with blue light comparing to 100R treatment, while *psbC*, that encodes for the CP43 core protein of PSII, was only significantly higher in 75R (Figure 6A,B). The gene coding for the large subunit of RuBisCo (*rbcL*) showed a progressive increase with the increase of blue light percentage, however 100B showed an upregulation comparable to 50R (Figure 6C). Finally, 100R showed the lowest expression of this gene. In infected plants, the expression of *psbA*, *psbC* and *rbcL* was generally reduced in comparison to healthy plants, except for 100 W and 100B where it was increased for all the three genes, and 100R where it was increased in *psbC* (Figure 6).

### 2.6. Hierarchical Clustering Analysis

**Figure 7:**
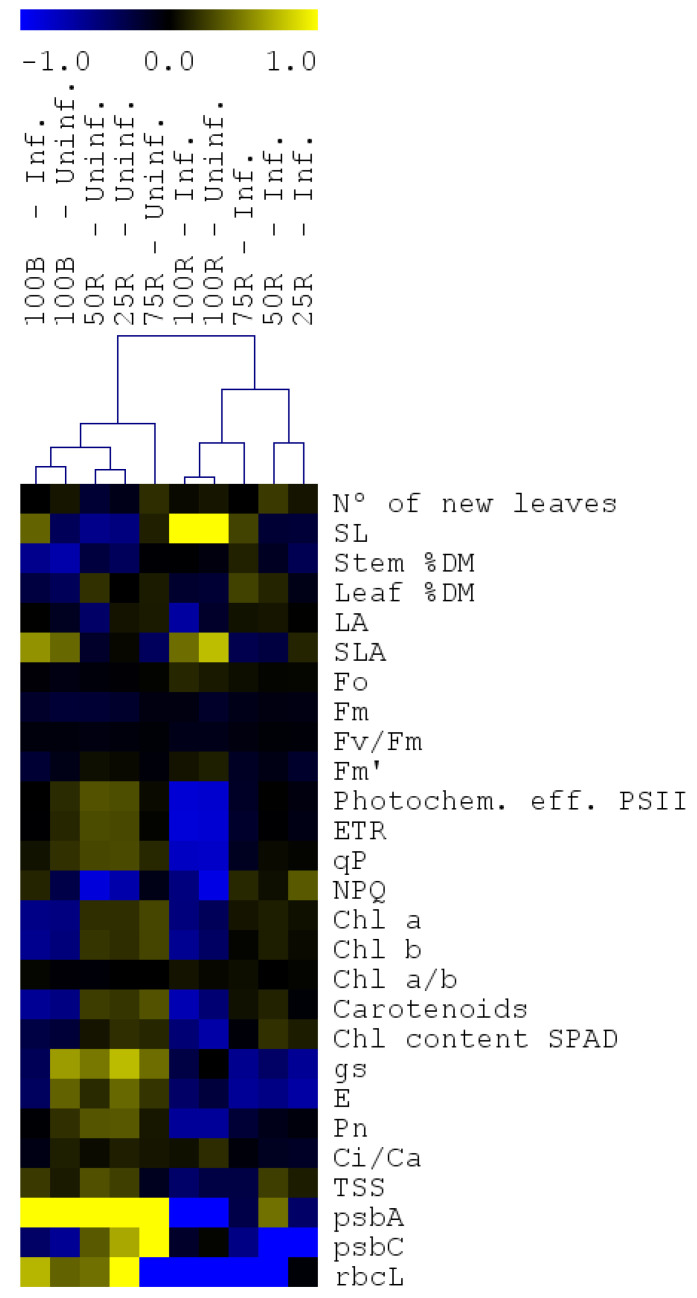
Hierarchical clustering of all parameters evaluated in the different light treatment. Clusters were generated using MeV with values normalized with the respective white light controls. Normalized values based on 100 W (uninfected or infected) data were used for hierarchical clustering based on Pearson’s distance metric. The color scale represents higher (yellow) or lower (blue) values with respect to the 100 W.

Hierarchical clustering of biometric and photosynthetic parameters revealed two main clusters (Figure 7). The first included all the uninfected plants except the 100R and the infected 100B. The second included all the infected plants except 100B treated ones and the uninfected 100R plants. Regarding the first cluster, most of biometric parameters showed a slight reduction in uninfected groups in comparison to 100 W, while in the corresponding infected groups a slight increase was observed. Chl a fluorescence-related parameters were not strongly different for uninfected and infected groups relative to the respective controls (100 W), while pigments, gas exchange-related parameters and gene-expression showed a general induction and neutral/repression effect in uninfected and infected groups, respectively. The sub-cluster comprising 100R uninfected and infected plants shows a predominance of negative effects of these treatments.

### 2.7. Effect of Light Treatments on Disease Development

**Figure 8:**
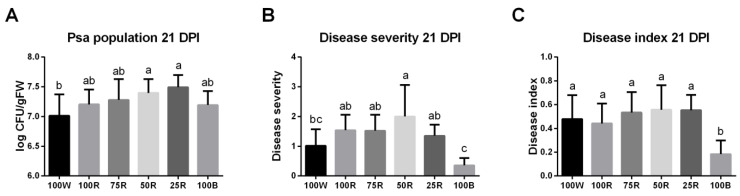
Effect of light quality on Psa population (**A**), Disease severity (**B**) and Disease index (**C**), 21 days post-inoculation (DPI). The values are presented as the mean of 3–5 biological replicates ± SE. Lowercase letters represent multiple comparisons of the light treatments. Mean values with the same letter are not significantly different (Fisher LSD, *p* < 0.05).

Symptom development and endophytic Psa populations were evaluated 21 days after the inoculation of *A. chinensis* var. deliciosa ‘Hayward’ plants grown under different light treatments. In comparison to control (100 W), the Psa population was significantly increased by 50R and 25R lights (Figure 8A). Moreover, red light generally tended to increase severity, and this increase become significant with 50R (Figure 8B). By contrast, while not affecting in planta bacterial growth, symptom development was significantly reduced by monochromatic blue light (100B) (Figure 8B,C; Supplementary Figure S2).

## 3. Discussion

### 3.1. Light and Photosynthesis in Kiwifruit Plants

Concerning the modulation of plant morphological features by light quality, our results pinpoint that red light increases etiolation and SLA, which is a typical ecological response associated to light-adaptation in shade-tolerant species, such as kiwifruit, evolved in undergrowth habitats [50]. Similar results have already been reported for plants exposed to red light, resulting in the so-called red-light syndrome [51]. In our study, the decrease in R:B ratio led to a decrease in stem and leaf %DM suggesting that the addition of blue light (or reduction of red light) leads to a decrease in dry mass content. Although blue light is often associated with a dwarfing effect on leaves, our study did not show any correlation between blue light percentage and LA, possibly because, being a shade-tolerant species, kiwifruit might be more adapted to lower R:B ratios in comparison to light-loving plants [52,53]. SLA showed also a slight correlation with blue light content, with an increase in blue light resulting in an increase in SLA. This finding is contrary to what was reported by [54], where the increase in blue light led to an increase in shoot dry mass and leaf mass per area (inversely proportional to SLA) in cucumber and tomato; increasing evidence however suggests that plant responses to light spectrum are species dependent [54,55] and follow an optimum function in RB ratio [27,53]. Both monochromatic light treatments presented a significantly lower leaf %DM and higher SLA, which might be due to a non-stimulation of cryptochrome and phototropin, in the case of 100R treatment, and an insufficient relative amount of active phytochrome that might be reached only in the presence of red light, in the case of 100B [56,57]. Zheng et al. [8] have further demonstrated that leaves treated with monochromatic red light were characterized by a lower thickness of palisade and mesophyll tissues when compared to plants exposed to white, blue or red-blue light treatments, which is another adaptation in the photosynthetic structures to optimize efficiency at low light levels [58,59].

Plants grown under monochromatic blue light generally exhibit higher photosynthetic capacity than those under monochromatic red light [20]. In this study, the higher F0 in 100R treated plants indicates that, even after prolonged dark periods, the amount of reaction centers that are able to receive electrons is lower than that in plants grown under any of the other light treatments. In line with this finding, 100R leaves showed the lowest F_v_/F_m_ ratio, a parameter which in healthy, non-stressed leaves generally shows values close to 0.8 [60] and is widely used as an indicator of photoinhibition or sustained photoprotection of PSII complexes [61]. In combination with higher F_0_ values in particular, a lower F_v_/F_m_ is understood to indicate the occurrence of photoinhibitory damage [60]. Regarding ΦPSII, data suggests that blue light, independently of the R:B ratio, is crucial to keep this parameter close to control. Indeed, in the absence of blue light this value was almost half of the corresponding value in 100 W; qP also showed a similar trend to ΦPSII. A possible hypothesis is that 100R treated plants might be showing a disordered chloroplast ultrastructure that could contribute to relatively low efficiency of light-dependent reactions [51] or an imbalance between degradation and replacement of D1 protein [20]. In fact, our results from the gene expression of *psbA* (encoding PSII D1 protein) showed a significant reduction in 100R in comparison to blue-light supplemented treatments. These results are also in line with those reported in Camellia sinensis [62], showing that the expression of most genes encoding these complexes (psa, psb, LHCA and LHCB) were downregulated in the red-light treatment, but upregulated by blue light. The dysfunctional photosynthesis in 100R-treated leaves was also evidenced in the results obtained for ETR and ΦPSII from rapid light curves, where the low ETR capacity was evident by reaching only about half the ETR of the other light treatments. This demonstrates that increasing the photon intensity could not compensate the deficiencies in the photosystem operational capacity for electron transport. The supplementation with 25% blue light (75R) was sufficient to have a photosystem similar to control, in accordance with [20] that demonstrated that blue light is more essential than red light to maintain PSI and PSII functionality and a good ETR capacity. In fact, the reduced ETR in 100R might be due to an impaired electron transport chain unable to keep the electrons transport to PSI, leading to a cyclic electron flow activation, with the return of the electrons from PSI to cytochrome b6f complex [20]. This would inhibit the electrons transport from PSII to PSI and, consequently, increase the dissipated energy in PSII, which goes in line with the lower ΦPSII observed in this experiment. Moreover, 100R treated plants presented a lower NPQ, the major photoprotective regulatory mechanism in higher plant thylakoid membranes, supporting the hypothesis that kiwifruit plants need at least a small amount of blue light to establish an efficient energy dissipation process, which ensures the photoprotection of the thylakoid [63]. NPQ reduces the concentration of chlorophyll excited states in PSII by the activation of a heat dissipation channel that facilitates NPQ as a major photo-protective response [64]. The impaired electron transport chain and the reduced NPQ may lead to the formation of reactive oxygen species, such as singlet oxygen, which can cause oxidative damage to several cellular components. Carotenoids are known to act as photoprotectors, receiving the excess of excited electrons from chlorophylls and preventing their reaction with molecular oxygen. However, carotenoids were reduced in 100R-treated plants which may explain the low ΦPSII and F_v_/F_m_ and higher F_0_, suggesting a degree of photodamage. Overall, 100R negatively influenced several parameters of photosynthesis, including photosynthetic pigments (chlorophylls and carotenoids), F_v_/F_m_, as well as ΦPSII, qP and NPQ. These results are further corroborated by the reduced ability of 100R treated plants to transport electrons even at higher PPFD, ability that is, instead, restored by supplementing blue light (Figure 4). 100B-treated plants, despite the slightly decreased F_v_/F_m_, did not show functional parameters such as qP or NPQ compromised nor the photosynthetic performance of the electron transport chain. Moreover, and in agreement with the literature, blue light (alone, but specially combined) did not reduce the levels of the majority of the photosynthetic pigments [21]. Overall, these results support the role of blue light in positively influencing chlorophyll biosynthesis and photosynthetic apparatus development [21,65].

Monochromatic red light is often associated with lower photosynthetic rates. In cucumber, for example, prolonged red light exposition impaired the photosynthetic capacity and led to unresponsive *g_s_*, while adding blue light to red light restored these parameters [51,52,53,54,55,56,57,58,59,60,61,62,63,64,65]. In kiwifruit, blue light treatment (alone or combined) increased *g_s_* and *E*, in accordance with previous findings where blue light directly stimulated stomatal opening through photoreceptors, leading to proton extrusion, membrane hyperpolarization and K^+^ and anion uptake mediating stomatal guard cells opening [66,67]. Blue light supplemented plants showed also a higher *Pn*, compared to the control, showing the benefits of the presence of blue light (even at low levels). 100R, on the contrary, showed the lowest levels of *Pn*. Furthermore, the high levels of C_i_/C_a_ in 100R treated plants corroborate the idea that the plants’ photosynthetic activity is reduced by the low ETR and consequently reduced ATP and NADPH production, rather than by stomatal closure in the absence of blue light. The low expression levels of the large subunit of RuBisCo (*rbcL*) represent another indicator that intercellular CO_2_ is not being used in the carboxylation phase of Calvin-Benson cycle. Lower photosynthetic rates, in turn, could explain the reduced level in TSS.

### 3.2. Light Quality, Psa and the Pathosystem

The use of light to control bacterial diseases is an emerging technique in modern agriculture that relies on the ability of light quality to modulate plant infection, either by conditioning pathogen virulence and/or by priming plant defenses [28,29,30,68].

In terms of biometric parameters, Psa infection significantly reduced shoot height and leaf surface area only under 100R light. In this condition, the plant is already significantly affected by the monochromatic light and may allocate the limited photoassimilates to the defense processes required to limit the colonization by the pathogen, thus further slowing down growth [46,69,70,71,72]. Plant infection is also commonly accompanied by decreases in chlorophyll content, down-regulation of light harvesting antenna proteins expression, decrease in the light harvesting ability, the activities of the photosystem II and I and down-regulation of photosynthesis-related genes [70,73,74]. Accordingly, the expression of *psbA* and *psbC* in kiwifruit was reduced by Psa infection in combined R:B treatments, although Chl a fluorescence and pigment content showed almost no significant differences.

Interestingly, kiwifruit plants subjected to monochromatic blue light (100B) showed a strong reduction in disease index and severity compared to the other light conditions, suggesting a protective capacity of this light treatment. Blue light is known to improve host plant resistance through different mechanisms, including for instance the accumulation of phenolic compounds, the induction of defense-related gene expression or the development of thicker leaves, ultimately reducing pathogen infection [75,76,77,78]. Thus, it would be worth to evaluate defense mechanism activation in this condition. However, kiwifruit infection did not lead to a reduction of photosynthesis or total soluble sugar content, often associated with defense induction, as mentioned above, and bacterial population was similar in all light treatments despite the induction of plant defenses usually characterized by pathogen growth restriction [79]. Hence, it cannot be ruled out that blue light indirectly improves kiwifruit resistance by reducing pathogen virulence, thus preventing defense mechanism inhibition and symptom development.

Stomata closure is part of a plant innate immune response to restrict bacterial invasion [80]. However, some pathogens have evolved mechanisms to overcome this defense and successfully colonize the plant tissues. Among virulence factors, Psa produces ethylene, which not only interferes with abscisic acid-mediated stomatal closure, but also antagonizes salicylic acid-induced responses, leaving the plant more susceptible to pathogen attack [34,81]. In blue light supplemented conditions, the net photosynthetic rate (Pn) was higher, and, thus, the intracellular redox state was mainly reductive due to the formation of NADPH, reduced ferredoxin and thioredoxin at the end of the photosynthetic electron transport chain, which is likely to be less favorable for ethylene production by Psa [34]. Accordingly, in plants subjected to blue-supplemented light, infection led to a slight reduction in stomatal conductance (g_s_), indicative of a more efficient stomatal closure that may contribute to kiwifruit resistance.

Light quality also modulates bacterial signaling regulating motility, attachment and virulence at the early stages of plant colonization by numerous plant pathogenic bacteria, including *Pseudomonas syringae*, *Agrobacterium tumefaciens* or *Xanthomonas campestris* [37,40,82]. While darkness is crucial for the growth of *Pseudomonas syringae* spp. and the increase of virulence traits such as motility that might be more favorably expressed at night to facilitate pathogen entry at dawn when stomata start opening [30,39,83], light, on the other hand, stimulates bacterial adhesion to leaves and biofilm formation [39,84]. In particular, in *P. syringae* pv. *tomato* DC3000, blue light is perceived by both the LOV-domain protein and the bacteriophytochrome BphP1 that regulate the expression of methyl chemotaxis protein required for Pto DC3000 full virulence [40,41,42,43,44,45,46,47,48,49,50,51,52,53,54,55,56,57,58,59,60,61,62,63,64,65,66,67,68,69,70,71,72,73,74,75,76,77,78,79,80,81,82,83,84,85]. Moreover, blue light perception by *Pseudomonas syringae* pv. *syringae* B728a LOV-HK protein positively regulates swarming motility by suppressing negative regulation by BphP1 [83]. On the other hand, in *Xanthomonas axonopodis* pv. *citri*, LOV-domain protein is involved in the control of host tissue damages caused by the pathogen to mitigate its virulence and avoid the occurrence of excessive tissue necroses that would be unfavorable for this hemibiotrophic pathogen [84]. Interestingly, the reduction of symptom development in plants infected with the *X. axonopodis* pv. *citri* lov mutant was associated with a normal bacterial growth compared with the wild-type strain, as observed in blue light-treated kiwifruit plants infected by Psa. Finally, *X. oryzae* pv. *oryzae* BphP controls virulence through the modulation of c-di-GMP [86], which has been proposed as an important second messenger for Psa virulence regulation [87]. Remarkably, in the most aggressive biovar of Psa (namely Psa3), used in this study, the LOV-domain protein homologue (WP_017683368.1) is constitutively expressed at higher levels compared to the biovars 1 and 2 [87], suggesting a possible role of blue light perception in Psa virulence. The investigation of the light wavelength effect on Psa transcription profiles as well as light-sensing protein functions in Psa deserve attention to unveil the molecular pathways involved in the regulation of Psa–*Actinidia* interaction in relation to light conditions.

This work shed light on the role of blue and red light in the photosynthetic performance of *A. chinensis* var. deliciosa plants. Monochromatic red light severely reduced PSII, ETR, Pn, TSS and the expression of *psbA* and *rbcL* genes. Both blue and red monochromatic lights led to a reduction in DW and pigments’ content. Red and blue light combinations, namely, 50R and 25R, showed the highest PSII efficiency and photosynthetic rate. However, these two light treatments were the least effective in controlling Psa endophytic population. Blue light, on the contrary, showed a great potential in controlling the disease development. Manipulating the light quality through LEDs (e.g., in nurseries) may thus be a powerful and sustainable tool to reduce Psa impact in kiwifruit. Concerning the practical application in orchard conditions, the knowledge acquired in this study allows for the optimization of the use of photoselective nets and plastic tunnels. In recent years, the use of photoselective nets has gained interest for kiwifruit cultivation due to their positive effects on fruit production and quality [88]; however, the effects on the plant physiology and response to pathogens have not been yet elucidated. Furthermore, the results of our experiments, despite being obtained on young plants, can be transferred to mature vines. In fact, at the whole canopy level new leaves are constantly produced (April to July), and they are the most exposed to sun light, being in the outer part of the canopy. Therefore, in light conditions similar to the ones imposed in our experiments, we would expect in adult plants similar results, at least on stomata opening, that is not known to be influenced by leaf age and also on disease symptoms development in agreement to I) the higher susceptibility of young leaves [42] and II) the hypothesis that symptom reduction is due to bacterial virulence reduction rather than plant resistance induction.

Further studies should aim at dissecting the influence of red and blue light on *Pseudomonas syringae* pv. actinidiae virulence and quorum sensing.

## 4. Materials and Methods

### 4.1. Plant Material and Growth Conditions

Two-month-old *Actinidia chinensis* var. *deliciosa* ‘Hayward’ plants (Dalmonte Vivai, Ravenna, Italy) grown in plastic pots (10 × 10 × 12 cm) were submitted to different artificial light treatments, tested in separated growing-compartments (0.24 m^2^ of surface, 0.15 m^3^ of volume and height-adjustable platforms). The experiment was performed in a climate-controlled growth chamber (temperature of 21 ± 1 °C and RH of 80 ± 6%), and growing compartments were insulated by metal panels. In each compartment, a fan system allowed a constant air circulation and the replacement of internal air, and dimmable LED lamps (Flygrow^®^, Flytech, Belluno, Italy) were installed.

Five different red and blue light treatments were adopted, providing red (R) and blue (B) light at different ratios, namely, 100%R (100R), 75%R:25%B (75R), 50%R:50%B (50R), 25%R:75%B (25R) and 100% B (100B) [27]. A control supplying a full spectrum of visible white light (100 W) was also included. The spectral distribution of each light treatment was measured using an illuminance spectrophotometer (CL-500A, Konica Minolta, Chiyoda, Tokyo, Japan) (Supplementary Figure S1). The same photosynthetic photon flux density (PPFD, 200 ± 8 μmol m^−2^ s^−1^) at the top of the canopy and photoperiod condition (16/8 light/dark) was used in all the treatments. A photosynthetic photon flux sensor (with equal sensitivity to red and blue radiation), model QSO (Apogee instruments, Logan, UT, USA), connected with a ProCheck handheld reader (Decagon Devices Inc., Pullman, WA, USA), was used to measure PPFD over the plant canopy. As plants were growing, the height of the compartments was increased, in order to maintain a constant distance between lamps and the top of the canopy, and, therefore, a constant PPFD. To avoid position effects and ensure a homogeneous distribution of the light across plants, these were moved clockwise, and the pot was rotated by 90º every two days. Nine plants were used per light treatment.

### 4.2. Plant Inoculation

After two weeks of adaptation to light treatments, plants were divided into two groups that were placed in physically-separated compartments of each light box. Five plants were inoculated (infected), and four were mock inoculated (uninfected) and used as control. For inoculation, Psa (CFBP 7286) was suspended in 10 mM MgSO_4_ to a concentration of 4 × 107 CFU mL^−1^. Plants were spray inoculated until run-off. Mock inoculated plants were sprayed with sterile 10 mM MgSO_4_. The relative humidity was raised to ~100% for the first 48h after inoculation and kept at 80 ± 6% thereafter. Disease severity and disease index were determined 21 days post inoculation (dpi) according to Cellini et al. [89].

### 4.3. Measurements of Plant Biometric Parameters

At the end of the experiment (21 dpi), the following parameters were measured on all the plants in each light treatment: number of newly grown leaves, stem length (SL), above ground (leaves plus stem and petioles) dry/fresh matter (DM/FM), leaf area (LA) and specific leaf area (SLA). LA was measured by a portable area meter (Li-3000, Li-COR Inc., Lincoln, NE, USA).

All leaves except the third leaf were immediately frozen in liquid nitrogen. Symptomatic and non-symptomatic leaves in the infected group were frozen separately, and all the subsequent analyses were performed only on the symptomatic leaves and the age-corresponding leaves on the uninfected group. The third leaf and stems were used for assessing dry matter (DM) after exposure to 65 °C for 72h. SLA and leaf DM/FM (%DM) were calculated for the third leaf only of each plant, where SLA is the ratio between LA and leaf DM.

### 4.4. Chlorophyll a Fluorescence and Pigments Quantification

Chlorophyll a (Chl a) fluorescence was measured on the fourth leaf of each plant, using a LI-6400 Portable Photosynthesis System (LI-COR Inc.). After each measurement, chlorophyll content was estimated with a portable chlorophyll meter SPAD-502 Plus (Konica Minolta Inc., Osaka, Japan), in the same spot where Chl a fluorescence was measured. Dark-adapted leaves (2 h dark adaptation) were used to measure minimum fluorescence (F0) under a weak modulated light and the maximum fluorescence (Fm) after applying a brief (1 s) saturating pulse of white light. The variable fluorescence (Fv = Fm − F0) and the maximum quantum efficiency of PSII [Fv/Fm = (Fm − F0)/Fm] were calculated. Light-adapted leaves were used to determine the steady-state fluorescence (Fs’), the maximum fluorescence (Fm’) and minimum fluorescence (F0’) under conditions of actinic light, after a 1 s saturating pulse and following brief dark relaxation (turning off the actinic source in the presence of a far-red >710-nm background light), respectively. Additionally, the PSII maximum efficiency (Fv’/Fm’), the photochemical efficiency of PSII (ΦPSII), electron transport rate (ETR), photochemical quenching (qP) and non-photochemical quenching (NPQ) were calculated according to Maxwell and Johnson [60] and Murchie and Lawson [90].

In addition to chlorophyll estimation by SPAD, photosynthetic pigments were also quantified. Leaves were ground in liquid nitrogen, and 100 mg were sampled for pigments extraction in acetone:50 mM Tris buffer (80:20, *v*/*v*) pH 7.8. For the extraction, samples were centrifuged for 10 min at 10,000× *g* at 4 °C. Chlorophyll a (Chl a), chlorophyll b (Chl b) and carotenoids (Car) contents were quantified by reading the absorbance at 470, 537, 647 and 663 nm in a multiplate reader Tecan Infinite ^®^ 200 Pro and calculated according to Sims and Gamon [91].

### 4.5. Rapid Light Curves (RLC)

The MONITORING-PAM fluorometer system (Heinz Walz GmbH, Effeltrich, Germany) was also used to calculate the ETR and ΦPSII from rapid light curves (RLC) recordings [92,93]. The RLC technique measures the fluorescence response to 10 different and increasing actinic irradiances of 10 s duration, each separated by a 0.8 s white saturating flash (2000–3000 µmol m^−2^ s^−1^). The RLC takes approximately 90 s and records PSII photochemical efficiency ΦPSII) as a function of PAR irradiance, from which electron transport rates (ETR) are estimated by the WinControl program (Walz GmbH, Effeltrich, Germany) under the assumptions of constant absorbance and energy partitioning.

### 4.6. Gas-Exchange Measurements and Soluble Sugars

Gas exchange was measured on the same leaf used for Chl a fluorescence analysis using a LI-6400 Portable Photosynthesis System (Li-COR Inc.). Measurements were done from 9 am until 12 pm. CO_2_ concentration was set to 400 ppm, which was close to the ambient CO_2_ concentration, with PPFD at 200 µmol m^−2^ s^−1^. Individual parameters were determined, including stomatal conductance (g_s_, mmol (H_2_O) m^−2^ s^−1^), transpiration rate (E, mmol (H_2_O) m^−2^ s^−1^), net photosynthetic rate (Pn, µmol (CO_2_) m^−2^ s^−1^) and intercellular CO_2_/ambient CO_2_ (C_i_/C_a_) [94].

Total soluble sugars (TSS) were quantified through the anthrone method by a Tecan Infinite ^®^ 200 Pro multiplate reader [95].

### 4.7. Photosynthesis-Related Gene Expression

The expression level of the ribulose-1,5-bisphosphate carboxylase/oxygenase large subunit (rbcL), and two core proteins of PSII (psbA and psbC), was determined on 6 plants (3 uninfected and 3 infected) for each light treatment. The primers used in this experiment (Table 1) were designed using Primer3Plus (http://www.bioinformatics.nl/cgi-bin/primer3plus/primer3plus.cgi/ accessed on 1 March 2022) software by using the sequence of GenBank Accession No. KC519936, AY323417 and AJ459488 as reference sequences, respectively. Their specificity was evaluated on cDNA and by melting curves analysis to exclude nonspecific amplification or primer dimers. RNA extraction was performed on 50–100 mg of leaf tissue and grinded in liquid nitrogen, using the SpectrumTM Plant Total RNA kit (Sigma-Aldrich). RNA quality and quantity were determined using a NeoDot UV/Vis Nano Spectrophotometer (Neo Biotech, Nanterre, France). One microgram of extracted RNA was used for cDNA synthesis using the PrimeScript RT Reagent Kit with gDNA Eraser (TaKaRa) and according to the manufacturer’s instructions. Real Time quantitative PCR analysis was performed with a dilution of cDNA (1:20), to which a master mix containing SYBR Green (Applied Biosystems, Foster City, CA, USA) and the primers of the genes of interest were added. The PCR reaction was conducted on an ABI PRISM Step One Plus system (Applied Biosystems), using the following cycling conditions: 95 °C held for 10 min followed by 40 cycles at 95 °C for 15 s and 58 °C for 1 min. The melting curve analysis ranged from 60 °C to 95 °C, with temperature increasing 0.6 °C. Each reaction was performed in 2 technical replicates, using actin [89] and gapdh [96] as constitutive genes. Amplification efficiency was calculated from raw data using LingReg PCR software [97]. The mean normalized expression (MNE)-value was calculated for each sample referred to the actin and gapdh expression according to the Simon equation [98]. Standard error (SE) values were calculated according to Pfaffl et al. [99].

### 4.8. Psa Quantification by Real-Time PCR (qPCR)

Symptoms were also correlated with the endophytic population of Psa quantified by qPCR according to Gallelli et al. [100]. DNA extraction was performed according to a modified Mercado et al. [101], on 100 mg of fresh leaf tissues ground in liquid N2. Modifications to Mercado et al. [101] were the following: (1) the pellet was resuspended in 650 µL of the washing buffer, to which 150 µL of 5 M NaCl, 100 µL of 10% N-laurylsarkosine, and 100 µL of 10% CTAB buffer was added and incubated at 65 °C for 30 min; (2) after organic/aqueous phases separation with dichloromethane: isoamyl alcohol (24:1), two volumes of cold isopropanol were added and incubated at −20 °C for 1 h; (3) the DNA pellet was dried in a speed vacuum to remove any remaining ethanol, and the DNA was dissolved in 50 µL of sterile water.

The DNA from pure cultures of Psa was harvested from the bacterial pellet obtained by centrifugation of 2 mL of an overnight grown culture. DNA was quantified with a NeoDot UV/Vis Nano Spectrophotometer (Neo Biotech) and diluted to 15 ng/µL for qPCR use. Psa was quantified according to Gallelli et al. [100]. The primers used were F (forward) 5′-GGTTTCGGACACCGCAGGTTCTACCGAG-3′ and R (reverse) 5′-CTTCCTGATCCCCGTTAC CCATCGAC-3′. Standard curves were prepared from 10-fold serial dilutions of bacterial genomic DNA, starting from 15 ng/µL (45 ng in the qPCR reaction). Linear regression curves were constructed by the threshold cycles (Ct) of each reaction against the log values of DNA concentrations. Cell quantity of Psa was calculated by interpolating the Ct values of the target samples to the standard curve included in each real-time PCR run. Since DNA extraction yield varies with the DNA extraction method and type of material used [102], the relationship between Ct values of the Psa DNA dilution series and the CFU corresponding to each dilution was achieved by considering Psa genome size (6.22 Mb) [103]. The amount of DNA may then be linked to CFU, and the Psa quantified as CFU.

Amplifications were carried out on an ABI PRISM Step One Plus system (Applied Biosystems). The detection system was based on SYBR Green dsDNA binding dye (Applied Biosystems), and the assays were carried out in a 10 µL reaction mixture with 3 µL of DNA. Cycling conditions were: 10 min at 95 °C, followed by 40 two-step cycles of 15 s at 95 °C and 1 min at 72 °C.

### 4.9. Hierarchical Clustering

For each experiment, raw values have been normalized to the respective white light control (in absence or presence of the pathogen) and imported as data matrices into MeV [104]. The data were adjusted as median center rows and clustered using the hierarchical clustering module. Sample trees were clustered with optimized sample leaf orders using the Pearson correlation and average linkage clustering. The trees were subsequently cut into clusters using a distance threshold (0.5 to 1) empirically adjusted to highlight the most relevant features of the trees.

### 4.10. Statistical Analysis

All statistical analyses were performed with GraphPad prism 6. The results are presented as mean with standard error (SE) bars. Significant differences were determined according to Fisher’s LSD multiple comparison tests at *p* < 0.05.

## Figures and Tables

**Table 1:** Primers used for gene expression study.

Gene		Primer Sequence
rubisco	F	ACATGGACAACTGTGTGGAC
	R	AGTTTCTTCTCCAGCAACGG
psbA	F	ATTCGTGAGCCTGTTTCTGG
	R	TCATCAACAGATGCCGCTTC
psbC	F	TGGTGGGGAAGTTATAGACACC
	R	ATACCGCCAAAGCCCAATAC

## Data Availability

Not applicable.

## Supplementary Materials

The supporting information can be downloaded at: https://www.mdpi.com/article/10.3390/ijms232113145/s1.
