# Supplementary material for: Red and Blue Light Differently Influence Actinidia chinensis Performance and Its Interaction with Pseudomonas syringae pv. Actinidiae
Source: Int J Mol Sci. 2022 Oct 29;23(21):13145. doi: 10.3390/ijms232113145 (PMC9658526; doi:10.3390/ijms232113145)
Supplement: Supplementary file 1 [file ijms-23-13145-s001.zip › ijms-1940713-supplementary.pdf]

Supplementary data

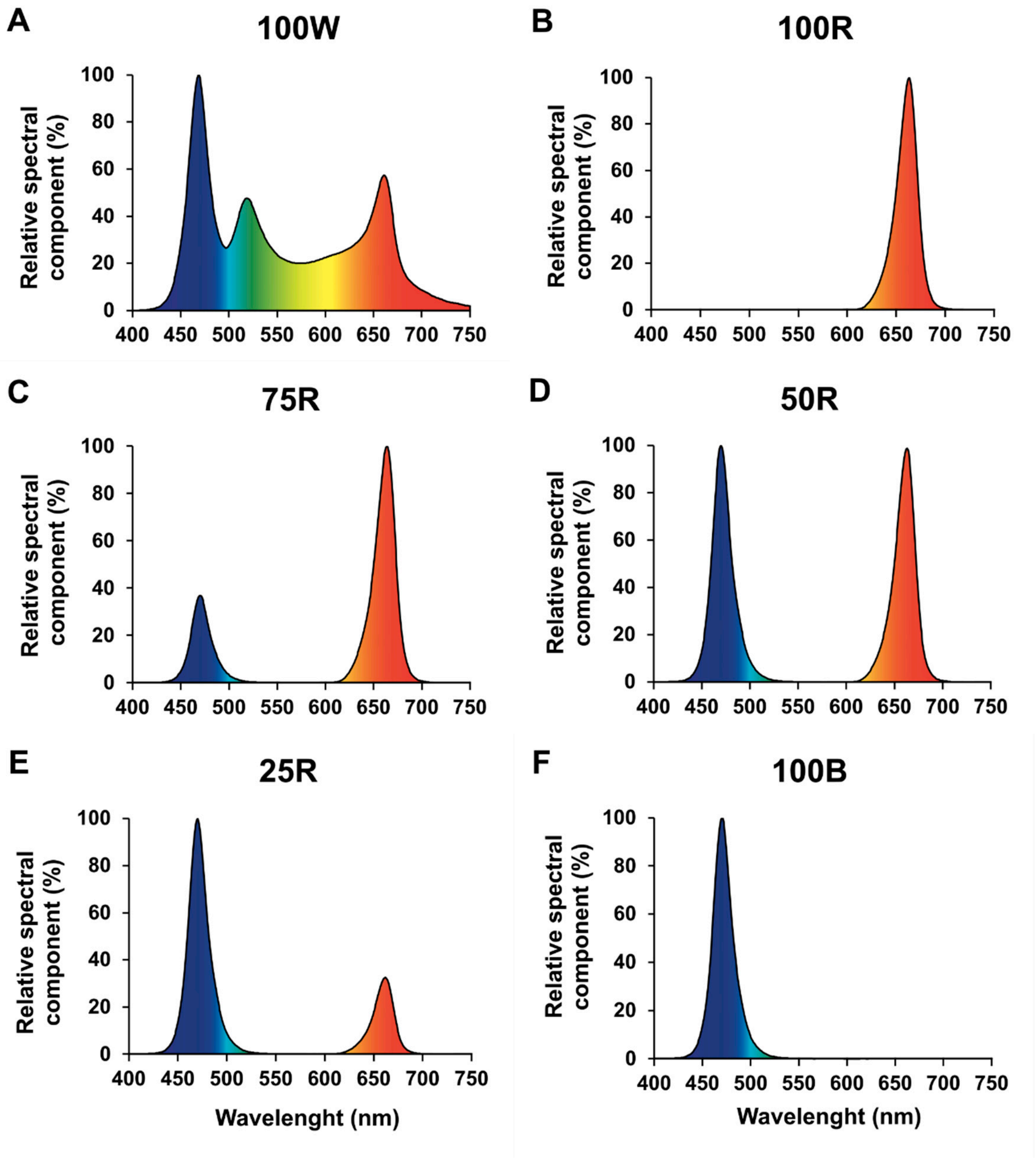

Figure S1- Wavelength spectra of the different lamps used in the experiments.

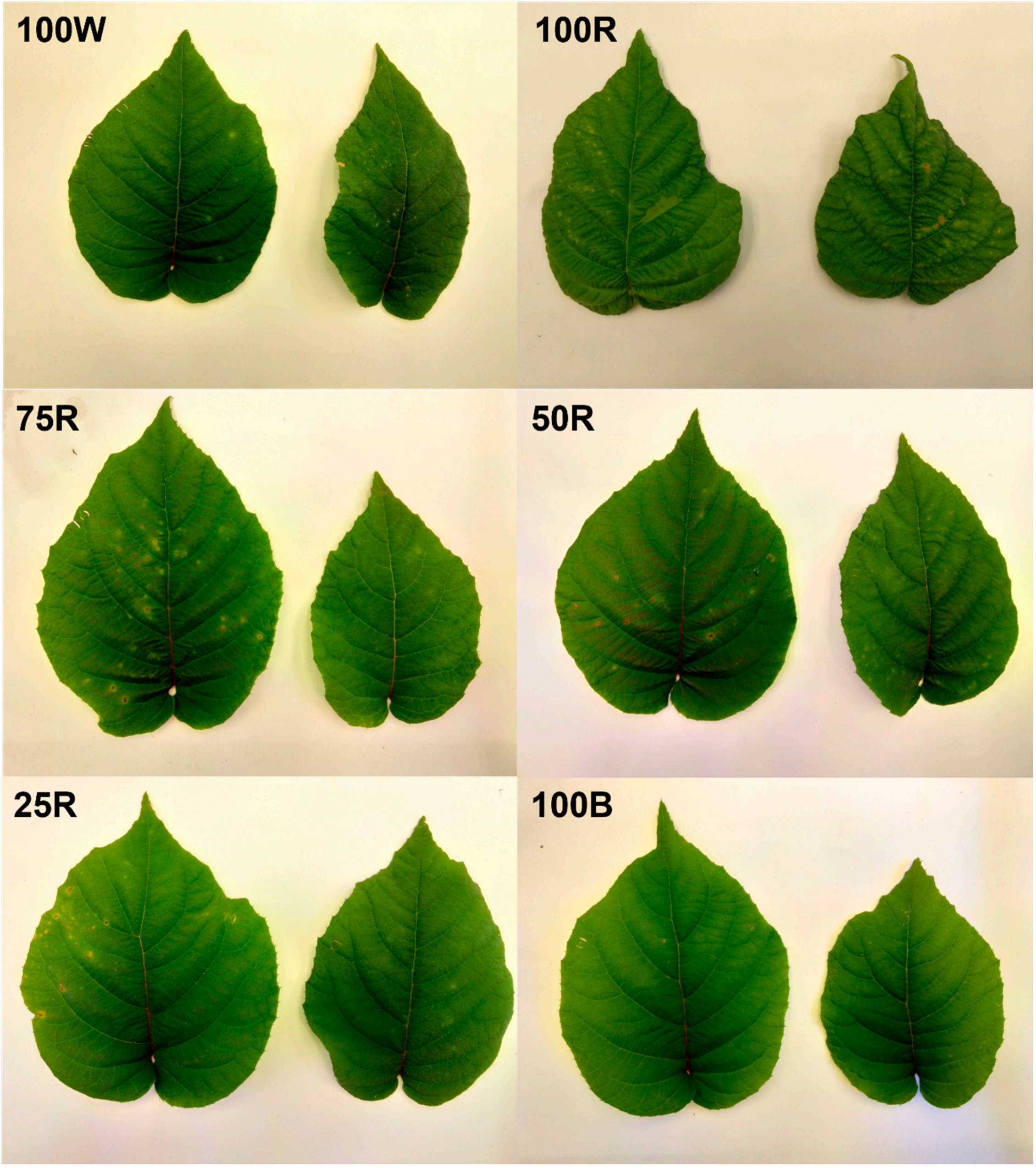

Figure S2 – Leaves from each light treatment showing symptoms.

Table S1 –Fisher LSD test on data from RLC (Fig. 4). Same letter are not significantly different (Fisher LSD, $p<0.05$). Multiple comparison analysis of φPSII of uninfected plants (A), ETR of uninfected plants (B), φPSII of infected plants (C) and ETR of infected plants (D).

A

PSII Uninf.	0	65	90	125	190	285	420	625	820
100W	a	a	bc	b	b	bc	bc	b	bc
100R	bc	b	c	b	b	c	c	c	c
75R	ab	a	ab	a	a	ab	ab	ab	ab
50R	c	a	ab	a	a	ab	ab	ab	ab
25R	c	a	ab	a	a	a	a	ab	a
100B	ab	a	ab	a	a	a	a	ab	a

B

ETR Uninf.	0	65	90	125	190	285	420	625	820
100W	a	a	a	a	a	a	a	a	a
100R	a	b	b	c	b	b	b	b	b
75R	a	a	a	ab	a	a	a	a	a
50R	a	a	a	b	a	a	a	a	a
25R	a	a	a	ab	a	a	a	a	a
100B	a	a	a	ab	a	a	a	a	a

C

PSII Inf.	0	65	90	125	190	285	420	625	820
100W	a	b	b	b	b	bc	bc	ab	ab
100R	b	c	c	c	c	c	c	b	b
75R	a	ab	b	b	ab	b	ab	ab	a
50R	a	ab	ab	ab	ab	ab	ab	a	a
25R	a	a	a	a	a	a	a	a	a
100B	a	ab	ab	ab	ab	ab	ab	a	a

D

ETR Inf.	0	65	90	125	190	285	420	625	820
100W	a	b	b	b	b	bc	bc	ab	ab
100R	a	c	c	c	c	c	c	b	b
75R	a	b	b	b	b	b	ab	ab	a
50R	a	ab	ab	ab	ab	ab	ab	a	a
25R	a	a	a	a	a	a	a	a	a
100B	a	ab	ab	ab	ab	ab	ab	a	a
